# Ex Vivo Major Histocompatibility Complex I Knockdown Prolongs Rejection-free Allograft Survival

**DOI:** 10.1097/GOX.0000000000001825

**Published:** 2018-06-11

**Authors:** Jessica B. Chang, William J. Rifkin, Marc A. Soares, April Duckworth, Nakul Rao, Yee Cheng Low, Jonathan P. Massie, Piul S. Rabbani, Pierre B. Saadeh, Daniel J. Ceradini

**Affiliations:** From the Hansjörg Wyss Department of Plastic Surgery, New York University Langone Health, New York, N.Y.

## Abstract

**Background:**

Widespread application of vascularized composite allotransplantation (VCA) is currently limited by the required lifelong systemic immunosuppression and its associated morbidity and mortality. This study evaluated the efficacy of ex vivo (after procurement but before transplantation) engineering of allografts using small interfering RNA to knockdown major histocompatibility complex I (MHC-I) and prolong rejection-free survival.

**Methods:**

Endothelial cells (ECs) were transfected with small interfering RNA targeted against MHC-I (siMHC-I) for all in vitro experiments. MHC-I surface expression and knockdown duration were evaluated using quantitative polymerase chain reaction (qPCR) and flow cytometry. After stimulating Lewis recipient cytotoxic lymphocytes (CTL) with allogeneic controls or siMHC-I–silenced ECs, lymphocyte proliferation, CTL-mediated and natural killer–mediated EC lysis were measured. Using an established VCA rat model, allografts were perfused ex vivo with siMHC-I before transplantation. Allografts were analyzed for MHC-I expression and clinical/histologic evidence of rejection.

**Results:**

Treatment with siMHC-I resulted in 80% knockdown of mRNA and 87% reduction in cell surface expression for up to 7 days in vitro (*P* < 0.05). Treatment of ECs with siMHC-I reduced lymphocyte proliferation and CTL-mediated cytotoxicity (77% and 50%, respectively, *P* < 0.01), without increasing natural killer–mediated cytotoxicity (*P* = 0.66). In a rat VCA model, ex vivo perfusion with siMHC-I reduced expression in all tissue compartments by at least 50% (*P* < 0.05). Knockdown prolonged rejection-free survival by 60% compared with nonsense-treated controls (*P* < 0.05).

**Conclusions:**

Ex vivo siMHC-I engineering can effectively modify allografts and significantly prolong rejection-free allograft survival. This novel approach may help reduce future systemic immunosuppression requirements in VCA recipients.

## Abstract 2

Supplemental Digital Content is available in the text.

## INTRODUCTION

Vascularized composite allotransplantation (VCA) has become a viable reconstructive option for severe defects not amenable to conventional reconstruction. Since 1998, over 107 upper extremity transplants,^1^ 39 face transplants,^2^ and numerous scalp, penis, and abdominal wall transplants have been performed worldwide,^3–5^ with promising short- and long-term outcomes. However, complications associated with the required lifelong immunosuppression can be severe. Renal toxicity, metabolic complications, and opportunistic infections are common, along with an increased risk of malignancy.^6–8^ Immunosuppression-related complications remain the major barrier to widespread application of VCA.

In the absence of immunosuppression, rejection inevitably occurs, causing allograft failure as early as 1 week posttransplantation. The acute rejection pathway begins with ischemic injury in the vascular bed. Naive lymphocytes encounter foreign antigens, travel to the lymph nodes, proliferate, reenter the vascular bed, and return to attack the tissue. The initial recognition of foreign antigens occurs at the molecular level via polymorphic major histocompatibility complex (MHC) cell surface expression.^9–11^ Donor MHC-I, which serves as a marker of self, is recognized as foreign by recipient CD8^+^ cytotoxic T-cells, initiating a cascade of antigen-specific activation and target cell lysis.^12,13^ Activated cytotoxic lymphocytes (CTL) cause direct lysis of allograft cells or produce inflammatory cytokines that recruit other immune cells, eventually causing allograft necrosis. In humans, a disparity between donor and recipient MHC class I, also known as human leukocyte antigens, is associated with an increased risk of rejection.^14–17^

To prevent rejection, multiple treatment approaches have been devised that interfere with T-cell and/or target cell adhesion, including T-cell vaccines,^18,19^ T-cell receptor antibodies.^20,21^ Human leukocyte antigen class II antibodies,^22^ CD4 antibodies,^23^ and blocking peptides that either occupy the T-cell receptor itself^24^ or obstruct co-stimulatory molecules critical for T-cell activation.^25,26^ However, these systemic treatments inevitably generate recipient immune responses, which can complicate therapy. Alternatively, the target cell itself may be modified before transplantation to prevent T-cell adhesion and activation, thus eliminating the need for systemic treatment of recipients and its associated complications.^27,28^ This approach has proved successful with the use of MHC-I monoclonal antibodies (mAb) and replication-incompetent adenoviral gene transfer.^29–31^

As a similar strategy, we attempted allograft modification via small interfering RNA (siRNA) targeted against MHC-I during the ex vivo period (after allograft procurement but before transplantation). We hypothesized that ex vivo reduction of MHC-I expression would prolong rejection-free survival via targeted inhibition of CTL-mediated rejection. This approach would not only target a critical mediator of rejection—the endothelial barrier—and help prevent rejection during the most vulnerable early postoperative period, but may also represent a potential target for future, more permanent gene therapies that would in turn decrease the need for systemic lifelong immunosuppression.

## MATERIALS AND METHODS

### Animals

All experiments were approved by the Institutional Animal Care and Use Committee of New York University School of Medicine and followed the National Institutes of Health *Guide for the Care and Use of Laboratory Animals.* A donor/recipient model was used for transplantation experiments. Adult male Brown-Norway and Lewis rats aged 12–14 weeks were used as donors and recipients, respectively. All animals were housed in an approved animal care center with a 12-hour light/dark cycle and provided standard rodent chow and water ad libitum.

### Cell Culture

Rat aortic endothelial cells (RAECs) (VEC Technologies, Rensselaer, N.Y.) were grown in endothelial cell growth medium-2 (Lonza, Basel, Switzerland), comprised of endothelial basal medium-2 supplemented with Single-Quots, 2% (v/v) fetal bovine serum (FBS), and 1% (v/v) penicillin-streptomycin. Cells were used between passages 3–7. All cells were incubated at 37°C in a humidified atmosphere containing 5% CO_2_.

### Purification of T-cells

A single cell suspension of rat splenocytes was obtained by dissociation of the spleen through a 70 μm strainer (BD Falcon, Franklin Lakes, N.J.) and washing of the debris with 1× cold phosphate-buffered saline (PBS). The splenocyte suspension was carefully layered on top of Histopaque 1077 (Sigma-Aldrich, St. Louis, Mo.) and centrifuged for 30 minutes at 400×G. The opaque interface was cautiously transferred at 300×G for 5 minutes and the pellet resuspended in magnetic-activated cell sorting (MACS) Buffering Solution (Miltenyi Biotec, San Diego, Calif.) supplemented with 0.2% (v/v) MACS bovine serum albumin (BSA) stock solution and incubated with 20 μL anti–T-Cell MicroBeads (Miltenyi Biotec) per 1.0 × 10^7^ total cells for 15 minutes on ice per the manufacturer’s instructions. After an additional washing step with MACS buffer solution, the pellet was resuspended in the same solution and applied to an LS magnetic column (Miltenyi Biotec). The effluent was discarded, the column removed, and the magnetically labeled cell fraction obtained by firmly and swiftly flushing the column with buffer.

### Purification of Natural Killer Cells

Splenocytes were obtained from Lewis rat spleens and subjected to 1× erythrocyte lysis buffer before filtration through an RPMI-1640 medium-soaked nylon wool column (Fisher Scientific, Waltham, Mass.) for depletion of B-cells as previously described.^32^ Following incubation for 1 hour at 37°C, the column was rinsed with RPMI-1640 medium containing 4% FBS and 1% antibiotic-antimycotic. The effluent was washed with ice cold MACS Buffering Solution supplemented with 0.2% (v/v) MACS BSA stock solution and incubated with 20 μL anti–T-Cell MicroBeads per 1.0 × 10^7^ total cells for 15 minutes on ice per the manufacturer’s instructions. After an additional washing step with MACS buffer solution, the pellet was resuspended in the same solution and applied to an LS magnetic column for depletion of T-cells. From this B- and T-cell–depleted effluent, natural killer (NK) cells were positively selected by labeling cells with a biotinylated anti-NKR-P1 mAb (Clone 10/78) for 30 minutes, washing twice, resuspending in buffer, and incubating with anti–T-Cell MicroBeads for 15 minutes on ice followed by *Streptavidin* staining antibodies for 5 minutes on ice. After washing, the resuspension was applied to LS columns and the effluent discarded. The column was removed from the MACS Separator and the fraction with magnetically labeled cells obtained by firmly and swiftly flushing the column with buffer. NK-cell purity was evaluated by flow cytometry.

### siRNA Design

MHC-I knockdown was achieved by transfection with siRNA-directed against conserved regions of MHC class I, specifically the RT1.Aa alpha-chain (Silencer siRNA, Applied Biosystems, Carlsbad, Calif.). As a control, nonsense siRNA (Silencer Select Negative Control No. 1 siRNA, Applied Biosystems) was used.

### In Vitro Transfection

In total, 3.0 × 10^4^ RAECs were seeded in 96-well plates and incubated for 24 hours. Medium was then replaced with serum-free starvation medium for 6 hours after which cells were transfected with either 20 or 40 nanomolar siRNA using Lipofectamine 2000 (Invitrogen, Grand Island, N.Y.). After 24 hours, starvation medium was replaced. Downregulation of MHC-I expression was assessed by flow cytometry and real-time quantitative polymerase chain reaction (qPCR).

### Flow Cytometry

The following mAbs were used for surface staining: fluorescein isothiocyanate (FITC)-conjugated anti-RT1A (Clone OX-18; BD Biosciences) and biotin-conjugated anti-NKR-P1A (Clone 10/78; BD Biosciences) for detection of endothelial MHC-I surface expression and of isolated NK cells, respectively. A total of 5.0 × 10^6^ cells were washed with 1× PBS cold staining buffer containing 1% BSA and 0.1% sodium azide, centrifuged, resuspended at a concentration of 1.0 × 10^6^ cells/mL staining buffer, and incubated for 30 minutes with the respective antibodies. Appropriate immunoglobulin G (IgG) isotype controls were included in all experiments. All samples were analyzed on a FACSCalibur flow cytometer using CellQuestPro Software (BD Biosciences).

### Activation of Cytotoxic CD8+ Lymphocytes, Mixed Lymphocyte Reaction, and Cytotoxicity Assay

Lewis rats were primed by the subcutaneous route with 200 μg of ovalbumin (OVA) (Fisher Scientific) in 200 μl of Complete Freund Adjuvant (Fisher Scientific). One week after systemic challenge, a booster consisting of 100 μg of OVA in 100μl of Complete Freund Adjuvant was applied through the same route. After an additional week, the spleen was removed aseptically, and a single cell suspension was prepared by mechanical dissociation methods, as described above.

Twenty-four hours after transfection, RAECs were exposed to a single 20-Gy dose of ionizing radiation. The following day, activated CD8^+^ T cells were added to target RAECs in the ratios of 1:1, 1:20, or 100:1 and incubated at 37°C for 4 hours. Cytotoxicity was determined using the Cytotox96 Cytotoxicity Assay (Fisher Scientific) kit. Briefly, Lysis Solution was added to each well, the plate centrifuged at 250 × g for 4 minutes, the supernatant transferred to a 96-well plate and incubated with Substrate Mix containing Assay Buffer for 30 minutes in the dark. Immediately after addition of Stop Solution, the plate was read at 490 nm according to manufacturer’s instructions.

### Natural Killer Cell Cytotoxicity

Twenty-four hours after irradiation, freshly harvested NK cells were added to target RAECs in ratios of 1000:1, 100:1, or 0.1:1 and incubated at 37°C for 4 hours. NK cytotoxicity was determined as above, following the instructions provided in the Cytotox96 Cytotoxicity Assay kit (Promega, Madison, Wis.).

### In Vivo Transfection

Superficial inferior epigastric (SIE) fasciocutaneous flaps were harvested as previously described.^33^ Rats were anesthetized with isoflurane (3% for induction and 0.5–2% for maintenance) mixed with oxygen. A 2-cm^2^ area was marked lateral to the groin, and the SIE flap was dissected following its vascular pedicle up to the inguinal ligament. The allograft, consisting of skin, adipose tissue, and its supporting vasculature, was detached and flushed with heparinized saline (100 U/mL) via the femoral artery. Once clear outflow was visualized from the femoral vein, the flap was perfused through the artery with 40 nM of siMHC-I or nonsense siRNA in Lipofectamine 2000, and incubated in 1× PBS at 37°C for 1 hour. The SIE vein and artery were anastomosed to their respective vessels in the recipient animal using 10-0 nylon sutures, and the skin closed with 6-0 monocryl sutures (Ethicon, Somerville, N.J.). Postoperative care included a 1-time bolus of normal saline (5 mL) and 7 units/kg/d of heparinized saline (100 U/mL) for 7 days. Recipients in both groups received a brief, 5-day course of cyclosporine (15 mg/kg, Sandimmune, Sigma Aldrich). Flaps were assessed daily for clinical signs of rejection, particularly the presence of any maculopapular erythematous rash, swelling, nodules or papules, and skin thickening and hardening.^34^ MHC-I mRNA expression was assessed with qPCR, and rejection-free survival was compared between the siMHC-I and control groups (n = 5 each).

### Quantitative Real-time PCR

For in vitro experiments, total RNA was extracted from cells using the RNeasy Mini Kit (Qiagen, Valencia, Calif.) on days 1, 3, 7, and 14 following transfection.

For in vivo experiments, allograft mRNA was isolated 72 hours after transplantation using the RNeasy Fibrous Tissue Midi Kit (Qiagen). The vascular pedicle, fat, and skin of the allograft were harvested, immersed in 2 mL of RLT buffer with β-mercaptoethanol, and immediately homogenized (Brinkmann Polytron Homogenizer PT 10–35). Total RNA was then isolated per the manufacturer’s instructions.

The mRNA was reverse transcribed using the QuantiTect Reverse Transcription kit (Qiagen). Quantitative real-time PCR was run on a ViiA 7 Real-Time PCR System (Applied Biosystems) with the SYBR Green PCR Master Mix (Applied Biosystems). Results were normalized to Glyceraldehyde 3-phosphate dehydrogenase (GAPDH).

### Immunohistochemistry

Samples from allografts perfused with siMHC-I or nonsense siRNA were harvested 72 hours posttransplant and snap-frozen in liquid nitrogen. FITC-labeled *Lycopersicon esculentum* lectin (Vector Laboratories, Burlingame, Calif.) was perfused through the SIE artery to stain all intact vasculature. siRNA was tagged with siGLO Red Transfection Indicator (Fisher Scientific) to confirm transfection. Functional endothelial beds were identified by colocalization of lectin stain and uptake of siRNA. Selected samples were mounted in DAPI (4´,6-diamidino-2-phenylindole) medium (Vector Laboratories), stained for MHC-I expression, and analyzed by immunofluorescence.

### Statistical Analysis

Data are expressed as mean ± SD. All experiments were performed at least in triplicate. Data distribution was analyzed, and statistical differences for different treatments were evaluated by analysis of variance, Kaplan-Meier survival curves, and Log-rank (Mantel-Cox) tests using SPSS version 21.0 (IBM Corp., Armonk, NY). Statistical significance was set at *P* < 0.05.

## RESULTS

### siMHC-I Transfection Reduces Endothelial Expression for up to 1 Week In Vitro

**Fig. 1.**
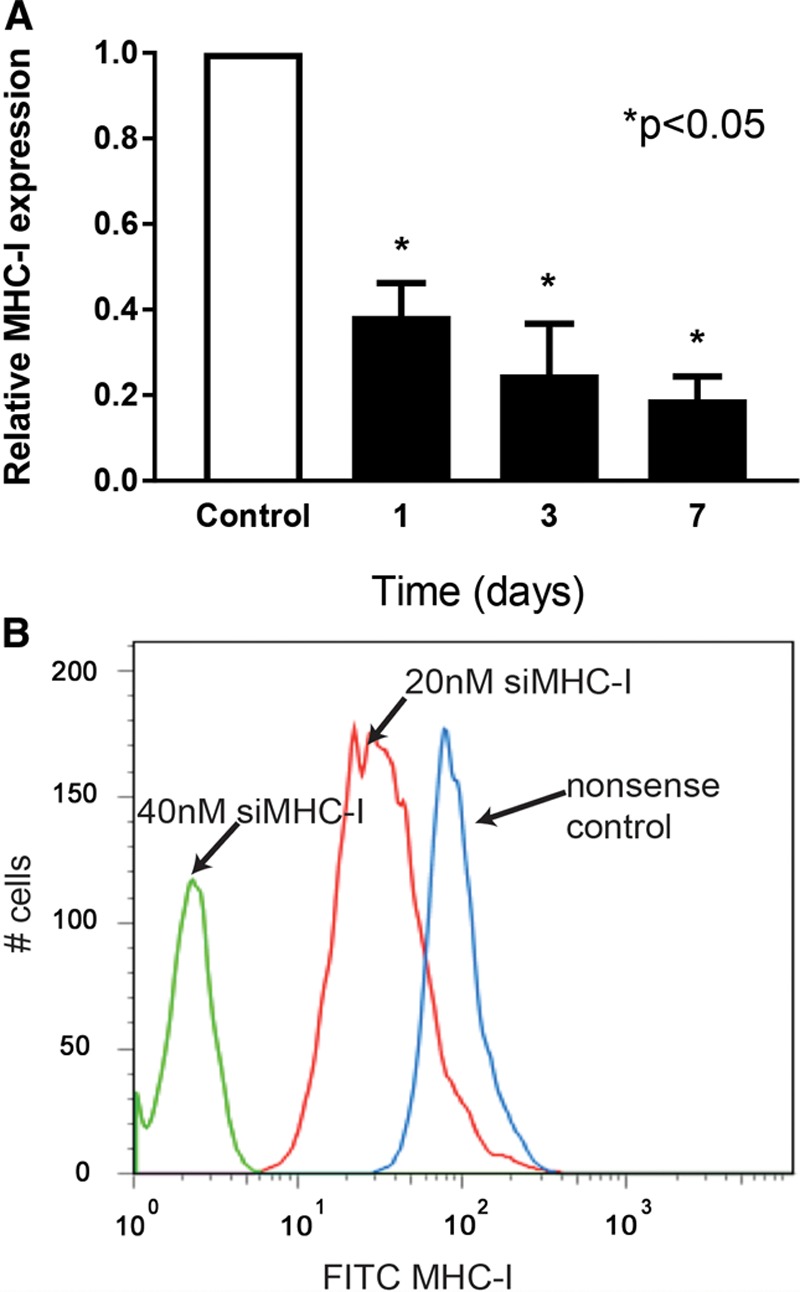
MHC-I knockdown in vitro. A, ECs silenced with a 1-time dose of 20 nanomolar siRNA against MHC-I at day 0 were collected at days 1, 3, and 7 posttreatment and compared with nonsense-treated controls using qPCR. Statistically significant reduction in relative MHC-I mRNA expression was observed at all time points as compared with control cells (*P* < 0.05; n = 3 for each). B, Knockdown of MHC-I on a protein level was analyzed on day 7 by flow cytometry and shown to be dose-dependent, as compared with nonsense controls (n = 3 for each).

Using previously established methods of siRNA transfection, in vitro knockdown of MHC-I in RAECs persisted up to 1 week at the RNA level (Fig. 1A). Knockdown was observed as early as 24 hours, with 62% ± 8% reduction of MHC-I mRNA expression, and an 81% ± 5% reduction on day 7 compared with controls (*P* < 0.05). At the protein level, FACS demonstrated a dose-dependent reduction in MHC-I cell-surface expression 7 days after treatment (Fig. 1B).

### MHC-I Silencing Reduces Lymphocyte Proliferation and Cell-mediated Cytotoxicity without Increasing Natural Killer Cell-mediated Cytotoxicity In Vitro

**Fig. 2.**
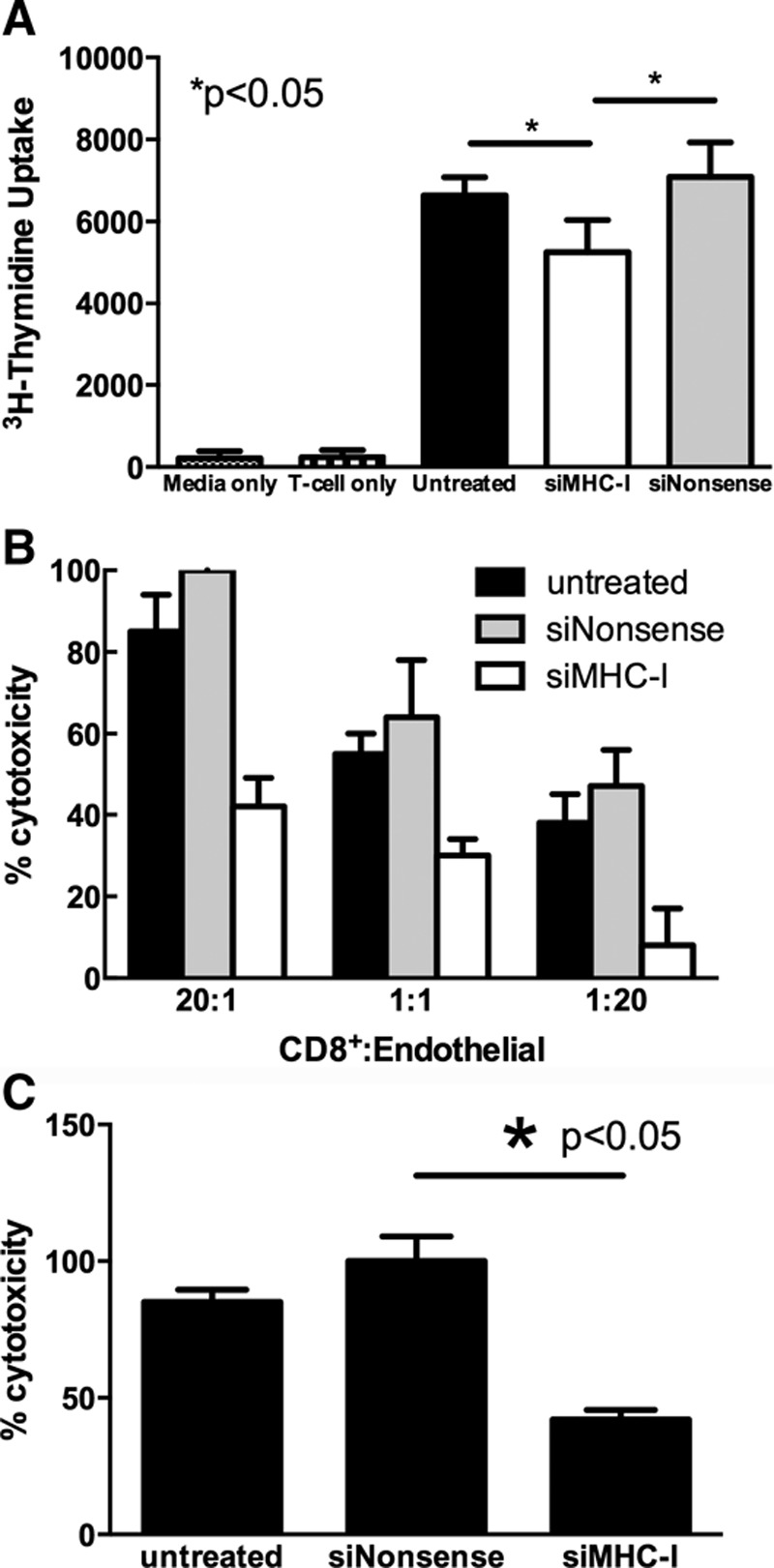
MHC-I silencing of endothelial cells reduces lymphocyte proliferation and CD8^+^ T cell cytotoxicity. A, MHC-I knockdown significantly reduced lymphocyte counts as compared with untreated and nonsense siRNA-treated RAECs 24 hours after transfection (*P* < 0.05; n = 3 for each). B, In target:effector ratios of 20:1, 1:1, and 1:20, MHC-I siRNA-treated RAECs demonstrated a marked reduction in cytotoxicity 24 hours after transfection (*P* < 0.05 for all; n = 3 for each). C, Target:effector ratio of 1:20 resulted in a 58% decrease in endothelial cell cytolysis compared with nonsense-treated controls 24 hours after transfection (*P* < 0.05; n = 3 for each).

A tritiated thymidine assay was used to measure lymphocyte proliferation in a mixed lymphocyte reaction. MHC-I knockdown decreased lymphocyte counts by 1,385 ± 445 and 1,838 ± 841 when compared with untreated and nonsense-treated RAECs, respectively (*P* < 0.05 for both; Fig. 2A).

The functional effect of silenced MHC-I expression was assessed in vitro via CTL activity. Activated CD8+ lymphocytes were cultured with MHC-I silenced in increasing target:effector ratios, resulting in a dose-dependent decrease in cytotoxicity (Fig. 2B). At a 1:20 CD8^+^ cell:endothelial cell ratio, MHC-I knockdown resulted in a 58% ± 11% reduction in CTL-cytotoxicity compared with nonsense-silenced controls (*P* < 0.05; Fig. 2C).

**Fig. 3.**
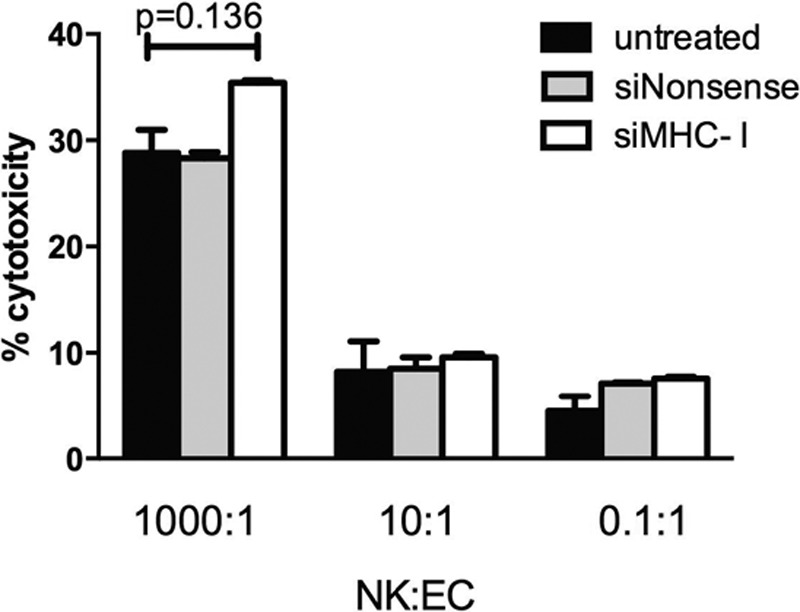
Natural killer cell-mediated cytotoxicity remains unchanged with MHC-I knockdown. NK:EC ratios of 1,000:1, 10:1, and 0.1:1 demonstrated no significant differences in NK-mediated cytotoxicity 24 hours after transfection (*P* > 0.1 for all; n = 3 for each).

NK cells were isolated and co-cultured with RAECs at ratios of 1,000:1, 10:1, and 0.1:1. There was no significant increase in endothelial cell toxicity for all ratios of NK cells and no change in NK-mediated cytolysis between untreated, siNonsense, and siMHC-I treatment groups (*P* > 0.1 for all; Fig. 3).

### Ex Vivo Silencing Reduces MHC-I Expression in Multiple Tissue Compartments

**Fig. 4.**
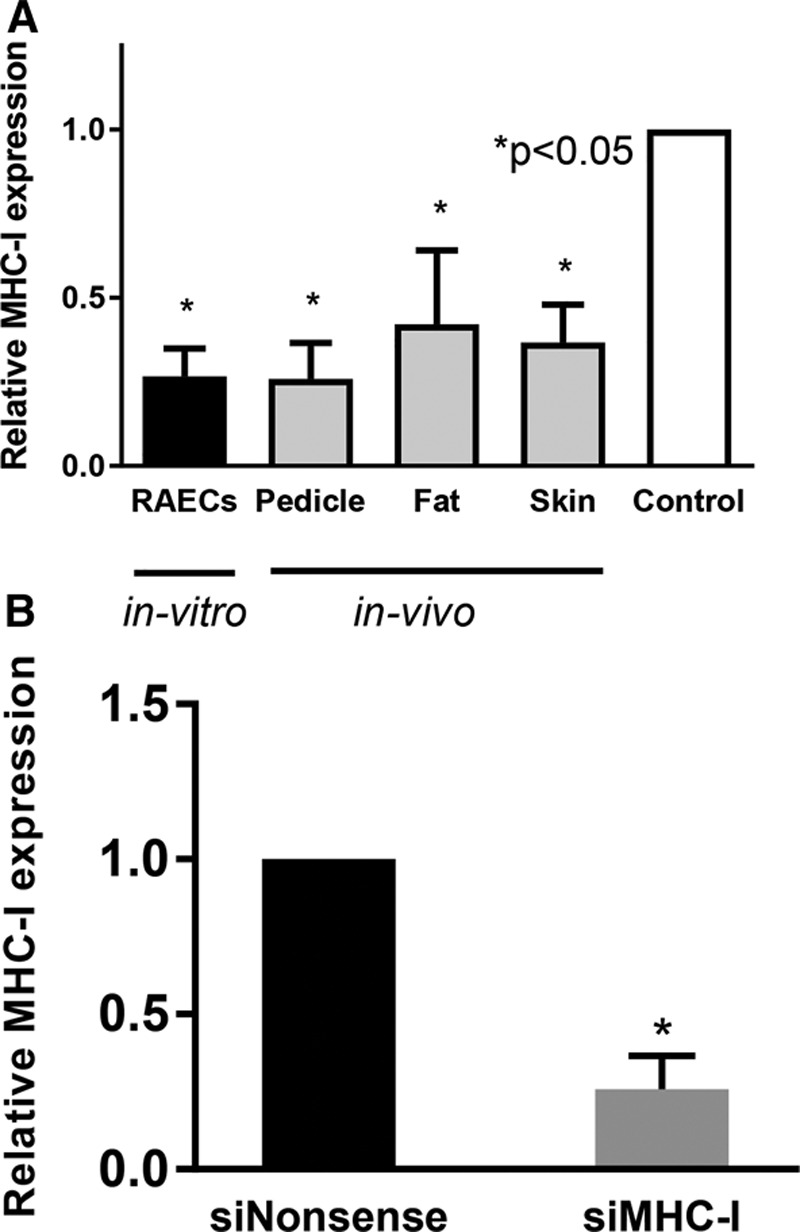
Ex vivo siMHC-I demonstrates effective knockdown of treated allografts. A, MHC-I mRNA expression is effectively reduced both in vitro and in vivo compared with nonsense-treated controls. Reduced expression was observed in vivo in multiple tissue compartments, including the vascular pedicle, fat, and skin, on postoperative day 3 (*P* < 0.05 for all; n = 3 for each). B, siMHC-I perfusion resulted in marked reduction of MHC-I expression in vascular tissue specifically on postoperative day 3 as compared with nonsense-treated controls (*P* < 0.05; n = 3 for each).

Perfusion of the allograft with MHC-I siRNA decreased MHC-I mRNA expression by 62% ± 11% in the pedicle, 58% ± 22% in the fat, and 63% ± 11% in the skin on postoperative day 3 (*P* < 0.05 for all; Fig. 4A). On postoperative day 7, MHC-I–treated pedicles demonstrated a 71% ± 14% knockdown in MHC-I expression compared with nonsense controls (*P* < 0.05; Fig. 4B).

### Ex Vivo Silencing Is Targeted Specifically to the Vasculature

**Fig. 5.**
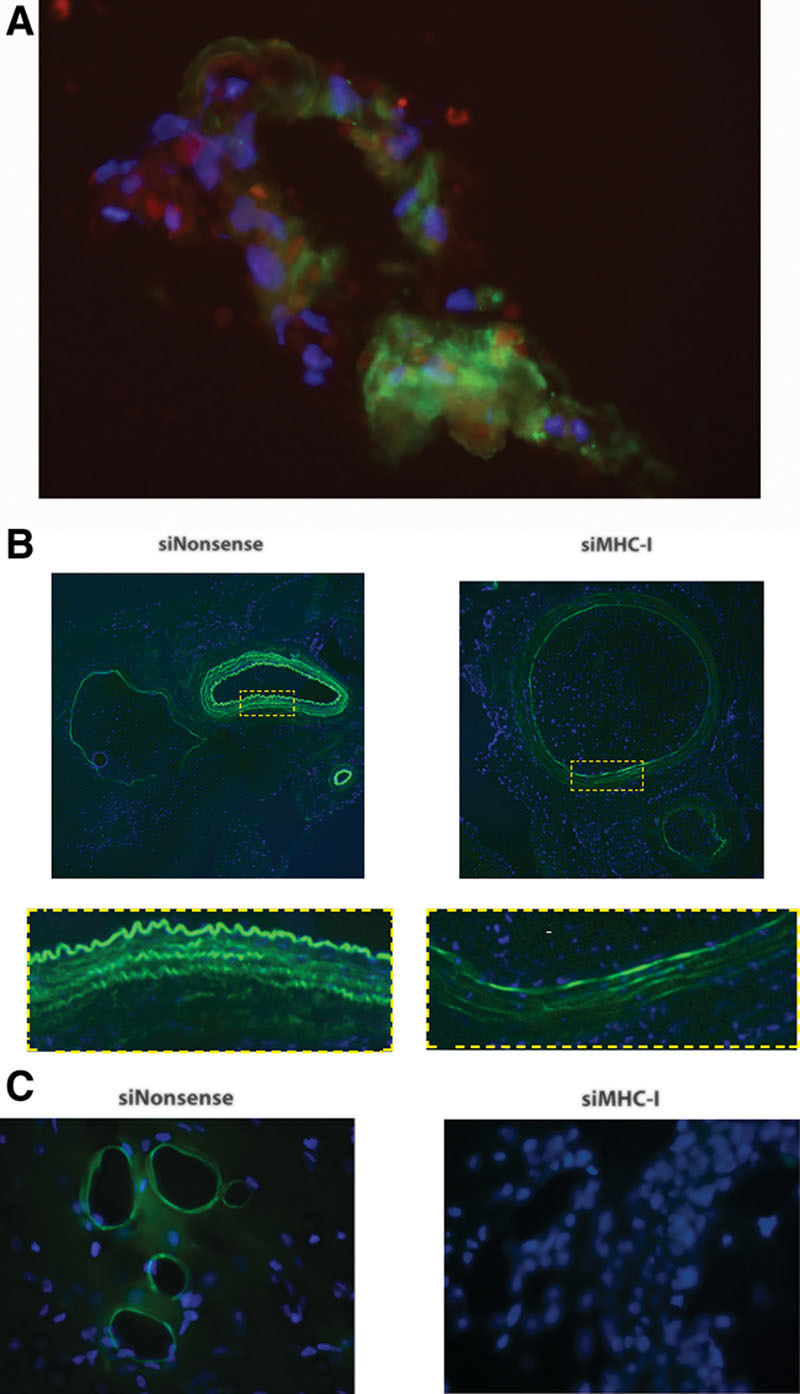
Immunohistochemistry of allograft vasculature on postoperative day 3. A, Flaps were co-perfused with FITC-lectin (green) to stain the intact vasculature and siGLO-labeled siRNA (red). Ex vivo perfused siRNA localizes to the perivascular space, where it exerts its effect on the endothelial barrier. B, Immunohistologic staining of MHC-I demonstrated marked reduction after ex vivo siMHC-I perfusion (right) as compared with siNonsense controls (left). MHC-I antigen is stained in green. Expression was reduced in multiple layers of the arterial and venous walls, but most prominently in the tunica intima, the innermost layer that is comprised of endothelial cells. C, MHC-I expression was completely masked at the microvascular level of the postcapillary venules.

Flaps co-perfused ex vivo with FITC lectin and DY-547-labeled siGLO demonstrated localization of siRNA to the allograft vasculature on immunohistochemistry (Fig. 5A). Ex vivo siMHC-I was shown to affect multiple layers of the arterial and vein walls, but most prominently affected the tunica intima, the innermost layer that is composed of endothelial cells (Fig. 5B). Further, treatment with siMHC-I completely masked MHC-I expression at the microvascular level in postcapillary venules (Fig. 5C).

### Ex Vivo MHC-I Silencing Prolongs Rejection-free Allograft Survival

**Fig. 6.**
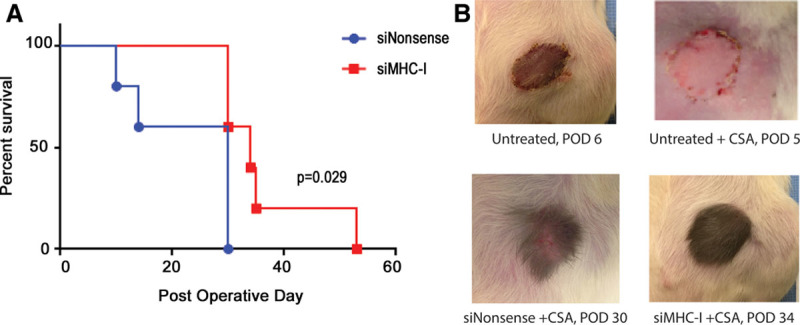
Kaplan-Meier in vivo survival curve. A, MHC-I silencing prolonged rejection-free survival of the allograft by 60% as compared with nonsense treated controls (Log-rank test: *P* = 0.029; n = 5 for each group). B, Untreated allografts without any systemic immunosuppression (top left) demonstrated clinical signs of acute rejection within the first week after transplant. However, addition of a brief 5-day course of CSA prevented early rejection (top right). Thus, ex vivo perfusion of nonsense and MHC-I-targeted siRNA was followed by a brief 5-day course of CSA. Control nonsense-treated flaps began exhibiting signs of rejection by postoperative day (POD) 14 (representative flap shown bottom left) in contrast to siMHC-I flaps, which took significantly longer to reject (representative flap shown bottom right). CSA, cyclosporine.

Following perfusion with either siMHC-I or nonsense siRNA, transplant recipients received a brief 5-day course of cyclosporine. The SIEA flap was then monitored clinically for signs of rejection, which was confirmed histologically. Ex vivo siMHC-I treatment prolonged rejection-free survival of the allograft by 60% compared with nonsense-treated controls (*P* < 0.05; Fig. 6). Importantly, no difference in wound healing was observed between groups, and laser Doppler imaging demonstrated equivalent revascularization rates between untreated, nonsense-treated, and siMHC-I-treated groups (**see figure, Supplemental Digital Content 1**, which displays laser Doppler imaging analysis, http://links.lww.com/PRSGO/A780). Additional allograft images are provided in Supplemental Digital Content 2 (see figure, **Supplemental Digital Content 2**, which displays the allograft images, http://links.lww.com/PRSGO/A781).

## DISCUSSION

VCA is the ideal method to restore delicate anatomy and function, but requires lifelong recipient immunosuppression. Current systemic immunosuppressive regimens carry the risk of organ failure, malignancy, and even death.^35^ The development of novel approaches to prevent rejection and reduce the immunosuppression burden would dramatically expand the potential applications of VCA. One such alternative approach is graft pretreatment, or modification of the allograft itself. Treatment of the graft after procurement but before transplantation would allow for timely and, importantly, localized targeting of the antigens that identify the donor as nonself. Mismatched MHC-I expressed on the microvascular endothelium is a critical mediator of rejection. Endothelial activation during allograft rejection is characterized by (1) expression of endothelial adhesion molecules in the rejecting allograft; (2) leukocyte migration into the tissue; (3) increased graft microvascular permeation by plasma proteins and fluid; and (4) edema accumulation in the allograft.^36^ Damage to the endothelial lining has been shown to be an early predictor of irreversible graft failure, and thus, protection of the endothelial barrier is critical. Friedman et al.^37^ demonstrated the pivotal role played by injury-induced inflammation in the VCA rejection pathway and present several possible targets for future intervention, such as the CXC3CL1 receptor.^37^

Several groups have previously targeted MHC-I to prevent rejection through different approaches. Faustman et al.^27,29^ used mAbs to mask donor MHC-I before transplantation of xenogeneic human pancreatic islets and liver and were able to promote long-term xenograft survival while eliminating the need for recipient immunosuppression. Beyer et al.^30^ employed adenoviral gene transfer of an anti-MHC I intrabody, which markedly reduced MHC-I surface expression and decreased cytotoxicity. Similarly, Furukawa et al.^31^ transduced vascular cells with an adenoviral vector expressing herpes simplex gene ICP47, which encodes a protein that binds to the host antigen-processing transporter and inhibits MHC-I formation. Both constitutive and inducible MHC-I expression were reduced, diminishing cytolysis by sensitized CTL.^31^

In the present study, we utilized siRNA transfection to silence MHC-I. RNA interference enables knockdown of endogenous genes by delivery of siRNA into cells, which triggers the destabilization and degradation of complementary mRNA sequences.^38,39^ Systemic, high-volume delivery of siRNA molecules has been shown previously to efficiently ablate target transcripts.^40^ However, to avoid systemic administration and any potential off-target effects, we instead perfused the allograft vasculature directly during the ex vivo period to achieve targeted and localized MHC-I knockdown.

With this approach, we demonstrated that knockdown of donor MHC-I expression reduces cell-mediated cytotoxicity, prolongs allograft survival, and may reduce the requirement for chronic immunosuppression. Our results show that siRNA-mediated posttranscriptional gene silencing effectively reduces expression of MHC-I both in vitro and in vivo. Importantly, MHC-I knockdown is functionally effective, reducing lymphocyte proliferation and CTL-mediated cytotoxicity, without an associated increase in NK cell-mediated endothelial cytolysis. In live animal models, ex vivo perfusion of allograft vasculature with siMHC-I significantly prolonged rejection-free survival compared with nonsense-treated controls. Since the inflammatory response in rejection is largely confined to the postcapillary venules,^41^ the dramatic reduction of MHC-I observed at this level on immunohistochemical analysis was particularly promising. However, a limitation of our study is its relatively small sample size, and larger cohorts are necessary moving forward to establish and confirm larger scale reproducibility of this technique. Additionally, the survival curves in our study touch at 30 days, suggesting that the effect—although significant—may not be that large. However, importantly there was a lack of rejection before 30 days within the MHC-1 silenced group compared with 40% of the control group that rejected within 2 weeks. Additionally, at 30 days (the point at which the curves touch), 100% of the control group displayed rejection, but 60% of the MHC-1 silenced group remained rejection-free, albeit for variable subsequent timelines. One potential explanation for the limited size of the effect may be the transient nature of the knockdown. This may explain in part the lack of early (< 30 day) rejection in the siMHC-1 group while the knockdown was present, with rejection ultimately occurring after the knockdown effect wore off and MHC-I expression levels normalized. In the future, longer-lasting (if not permanent) knockdown may result in an even larger effect.

The results of this study demonstrate that allografts can be effectively engineered with ex vivo perfusion of siMHC-I, resulting in localized and targeted reduction of foreign allograft recognition and a more tolerogenic phenotype. This serves as an important proof-of-concept for our novel technique of ex vivo allograft modification^42^ (specifically genetic modification of the allograft vasculature) and demonstrates the value of MHC-1 as a target for gene knockdown. However, the effects of siRNA are inherently transient, and the next challenge is to design a method to achieve longer lasting—if not permanent—allograft modification. This novel therapeutic approach to promoting allograft tolerance and preventing rejection is easily translatable, clinically feasible, and may ultimately reduce chronic immunosuppression requirements in VCA recipients.

## Supplementary Material
