# Supplementary figures and images for: Ex Vivo Major Histocompatibility Complex I Knockdown Prolongs Rejection-free Allograft Survival
Source: Plast Reconstr Surg Glob Open. 2018 Jun 11;6(6):e1825. doi: 10.1097/GOX.0000000000001825 (PMC6157929; doi:10.1097/GOX.0000000000001825)

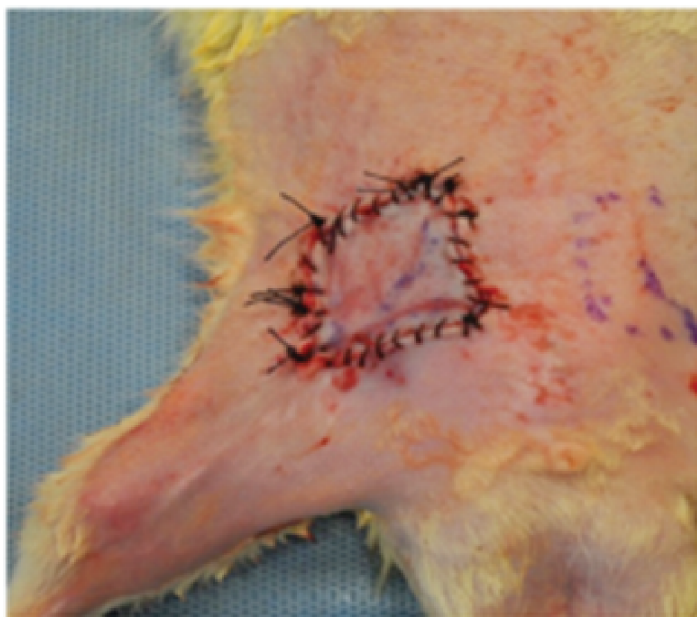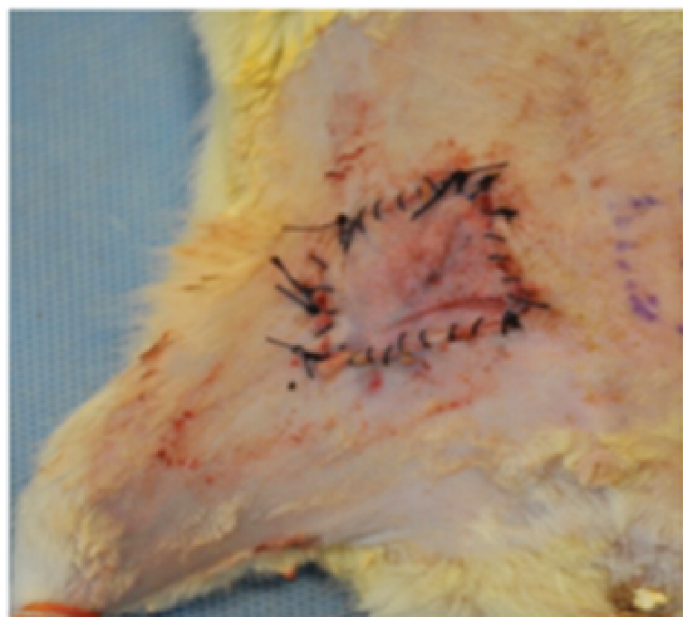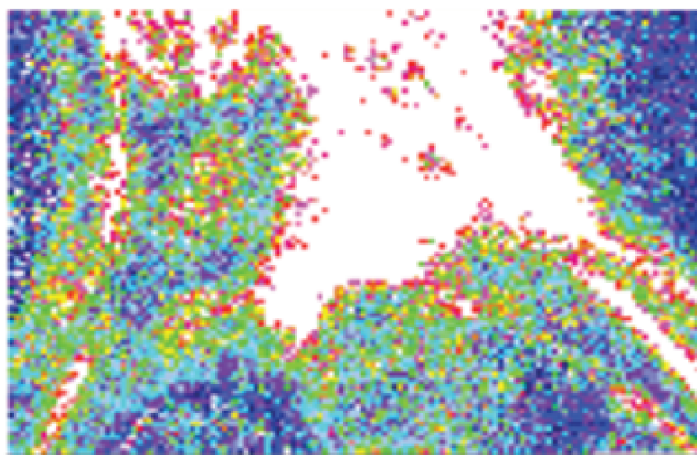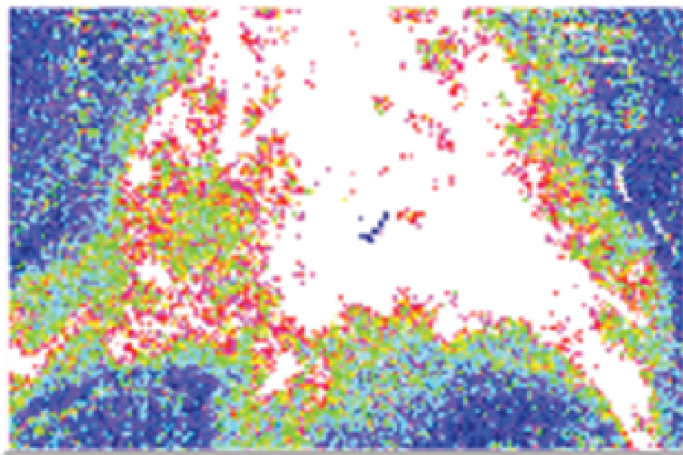

Supplement: Supplementary file 1 [file gox-6-e1825-s001.pdf]

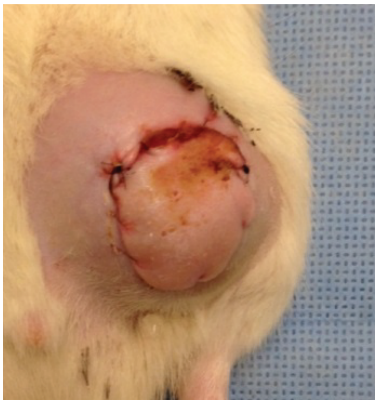

POD 1

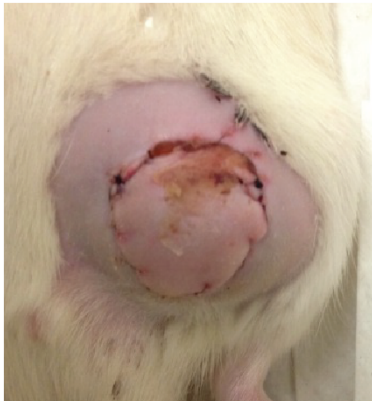

POD 5

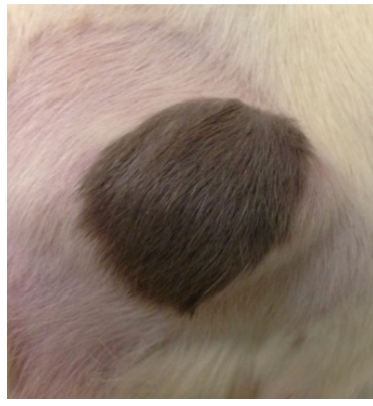

POD 28

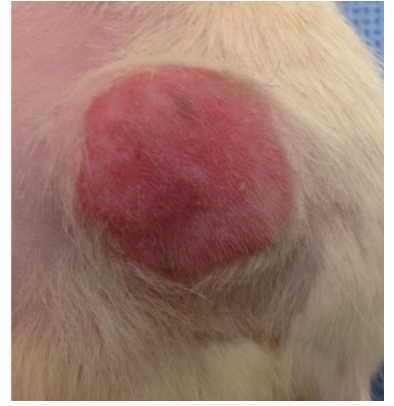

POD 35

Supplement: Supplementary file 2 [file gox-6-e1825-s002.pdf]
